# Insights into the Dynamics and Binding of Two Polyprotein Substrate Cleavage Points in the Context of the SARS-CoV-2 Main and Papain-like Proteases

**DOI:** 10.3390/molecules27238251

**Published:** 2022-11-26

**Authors:** Zainab Kemi Sanusi, Kevin Alan Lobb

**Affiliations:** 1 Department of Chemistry, Rhodes University, Makhanda 6140, South Africa; 2 Research Unit in Bioinformatics (RUBi), Rhodes University, Makhanda 6140, South Africa

**Keywords:** SARS-CoV-2 3CLpro, PLpro, protein substrate, molecular dynamics, molecular docking

## Abstract

It is well known that vital enzymes in the replication process of the coronavirus are the SARS-CoV-2 PLpro and SARS-CoV-2 3CLpro, both of which are important targets in the search for anti-coronavirus agents. These two enzymes are responsible for cleavage at various polyprotein sites in the SARS-CoV-2 lifecycle. Herein, the dynamics of the polyprotein cleavage sequences for the boundary between non-structural proteins Nsp1 and Nsp2 (CS1) and between Nsp2 and Nsp3 (CS2) in complex with both the papain-like protein PLpro and the main protease 3CLpro were explored using computational methods. The post dynamics analysis reveals that CS1 and CS2 both have greater stability when complexed with PLpro. Of these two, greater stability is observed for the CS1–PLpro complex, while destabilization resulting in loss of CS2 from the PLpro active site is observed for CS2-PLpro, suggesting the rate of exchange by the papain-like protease is faster for CS2 compared to CS1. On the other hand, the 3CLpro main protease also reveals stability for CS1 suggesting that the main protease could also play a potential role in the cleavage at point CS1. However, destabilization occurs early in the simulation for the complex CLpro–CS2 suggesting a poor interaction and non-plausible protease cleavage of the polyprotein at CS2 by the main protease. These findings could be used as a guide in the development and design of potent COVID-19 antiviral inhibitors that mimic the CS1 cleavage site.

## 1. Introduction

The severe acute respiratory syndrome—coronavirus 2 (SARS-CoV-2) belongs to the family of β-coronaviruses, and it is a causative agent of the novel coronavirus disease 2019 (COVID-19) [1,2,3]. For over two decades now, β-coronaviruses (β-CoVs) have caused three epidemics, namely SARS-CoV in 2002, MERS-CoV in 2013 and recently SARS-CoV-2 in 2019 [4,5]. The SARS-CoV-2 virus epidemic, which originated in Wuhan, China, and quickly spread throughout the world, was declared a pandemic by the World Health Organization (WHO) and has resulted in more than 6 million deaths globally as of present [6]. COVID-19 mainly affects the respiratory system and causes a flu-like illness with symptoms such as fever, cough and, in more acute cases, difficulty in breathing. The mortality rate is high in older (>60 years) individuals and people with underlying conditions [7,8]. COVID-19 is not limited to respiratory distress and failure; the virus is now known to emerge as a systemic inflammation that leads to severe cardiac injury, heart failure, sepsis and multi-organ dysfunction in individuals at high risk [3,9].

Tremendous work/research has been conducted by scientists all over the world to study the symptoms and risk factors for this disease, as well as understand the fundamental mechanism of the SARS-CoV-2 virus [8,10,11,12]. Currently, a few vaccines such as the Pfizer-BioNTech, Moderna and Johnson & Johnson vaccines are approved by the Food and Drug Administration (FDA), while Remdesivir [13] is the only antiviral drug approved by FDA [14,15]. Even though immunity can be achieved by taking the available vaccines, transmission of the virus is still possible; hence, wearing of masks, frequent washing of hands and social distancing are nevertheless mandated. Owing to the rapid infection and mortality rate, as well as emergence of new mutations of the virus, the COVID-19 virus does not seem to be disappearing. Hence, more effort is required in the elucidation of the pathogen’s strategies in search for more potential and effective therapeutic drugs.

**Figure 1:**
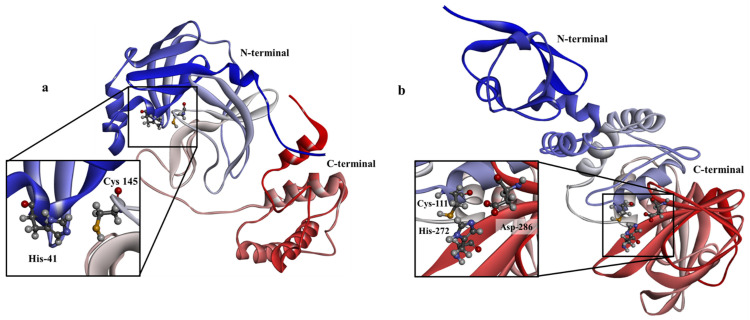
Three-dimensional structure of SARS-CoV-2 (**a**) main protease (3CLpro, PDB ID: 6XA4) [18] and (**b**) papain-like protease (PLpro, PDB ID: 6WX4) [19] showing the active site and essential domain within the enzyme (rendered using BIOVIA Discovery Studio [20]).

The SARS-CoV-2 is an RNA-based genome and infects the human host cells by binding to the cell surface of ACE2 through the receptor-binding domain (RBD) of its S protein [2,16]. A detailed insight into SARS-CoV-2 pathobiology/pathophysiology is still being developed, although the virus genome sequence is available in NCBI database (NC_045512.2) [17] and several possible antiviral targets have been identified [2,5,8]. These include the RNA-dependent RNA polymerase (RDRp), trans-membrane protease serine 2 (TMPRSS2), spike protein (S protein), angiotensin AT2 receptor (AT2), angiotensin-converting enzyme 2 (ACE2), papain-like protein (PLpro) and the main protease (3CLpro) [2,5,8]. The 3D structure of the SARS-CoV-2 main protease is illustrated in Figure 1.

The coronavirus main protein, also known as the chymotrypsin-like cysteine protease (3CLpro) [21], is a cysteine protease with a catalytic dyad (Cys145/His41) active site [12,17]. Like other cysteine proteases [22], the SARS-CoV-2 3CLpro cleaves its target using this dyad [12,21]. Other residues (Thr25, Thr26, Leu27, Ser46, Met49, Tyr54, Phe140, Leu141, Asn142, Gly143, His163, Met165, Glu166, Leu167, Pro168, Phe185, Asp187, Gln189, Thr190, Ala191 and Gln192) within the active site have been recognized as substrate binding residues and therefore play a significant role in the effective development of SARS-CoV-2 3CLpro inhibitors [23,24].

Both proteases (PLpro and 3CLpro) play a crucial role in viral polyprotein processing of the polyproteins that are translated from viral RNA during the replication and release of new SARS-CoV-2 virions [5,8]. Previous studies have shown that the papain-like protease (PLpro) recognizes and cleaves the viral polyproteins (pp1a and pp1ab) at the N-terminal to produce three non-structural protein (Nsp1, Nsp2 and Nsp3) [23,25,26], while the 3CLpro also recognizes and cleaves the viral polyprotein at the C-terminal and produces at least 11 new individual mature non-structural proteins. This is true throughout the wider coronavirus family, and it is observed that both protases are well conserved [5,8,27]. The specificity in cleaving polyproteins at points after Gly and after Gln residues is another conserved characteristic of the SARS-CoV-2 PLpro [28] and SARS-CoV-2 3CLpro [29] enzymes, respectively, which is also observed in other coronavirus proteases but not in human enzymes [27] (Table 1). Preventing the activity of these proteases would inhibit the lifecycle of the virus [5,23].

In the literature, there has been development of several competitive non-covalent inhibitors of PLpro from analogs of protein substrates of PLpro which consists of Leu-X-Gly-Gly amino acid peptides [23]. It is, therefore, necessary to understand how PLpro recognizes and cleaves the protein substrate (CS1 and CS2) and also if there is any analogous behavior by the 3CLpro enzyme. This is possible through computer simulations with the availability of solved X-ray structures [27,30,31] of the SARS-CoV-2 3CLpro and PLpro enzymes (Figure 1).

The 3CLpro is a primary target in the search for agents to treat the novel coronavirus infection [5]. In this study, we aim to investigate the dynamics of the structural properties and substrate binding landscape of the 3CLpro complex with the protein substrates (CS1 and CS2) [5], and this is compared with the corresponding PLpro complexes. Herein, a long-timescale molecular dynamics (MD) simulation was performed to provide adequate information on the complex dynamics. This was achieved by first performing molecular docking to generate both 3CLpro–CS1 and 3CLpro–CS2 complexes, as well as the PLpro–CS1 and PLpro–CS2 complexes. Post dynamics analysis, including root-mean-square deviation and fluctuation (RMSD and RMSF), the radius of gyration (RoG), dynamic cross-correlation matrices (DCCMs), hydrogen bonding (HB) and binding free energy calculations, was carried out. The findings demonstrate the conformational and structural features of the 3CLpro in complex with the studied protein substrates in comparison with the corresponding PLpro protease complexes. This could assist in the development of new inhibitors with improved and additional selective activity.

## 2. Results and Discussion

### 2.1. Docking Validation

The objective of re-docking is to validate the docking procedure, and this was achieved firstly by simply removing the co-crystallized inhibitors from PDB and saving them separately as new inhibitors in PDB format. The co-crystallized inhibitors were then re-docked into the active sites of both proteases (3CLpro and PLpro) using AutoDock Vina 1.2.0 [32], and the same protocol as described previously including search parameters was applied in order to ensure that the inhibitors bind precisely to the active sites.

**Figure 2:**
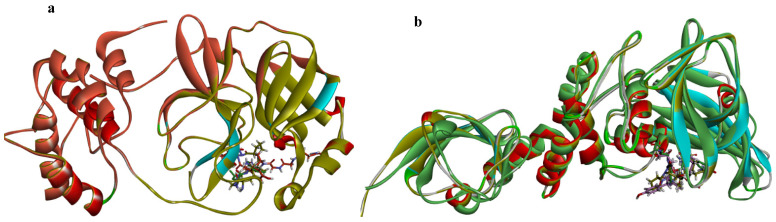
(**a**) Three-dimensional representation of the superimposed SARS-CoV-2 3CLpro (6XA4, blue) from PDB, 3CLpro–CS1(light brown) and 3CLpro–CS2 (red). (**b**) Superimposed SARS-CoV-2 PLpro (6WX4, blue) from PDB, PLpro–CS1(light brown) and PLpro–CS2 (red) to validate docking binding at the active site of the proteases.

The re-docked complexes were later superimposed using BIOVIA Discovery Studio [20], first on the native co-crystallized 6XA43 and 6WX4 complexes from PDB. A root-mean-square deviation (RMSD) of less than 1 Å was observed, respectively, validating the docking procedure. Subsequently, the native complexes were also superimposed on the 3CLpro–CS1/CS2 and PLpro–CS1/CS2, making a total of six superimposed amino peptide residues (Table 2 and Figure 2). This proves to an extent the validity and appreciable efficiency of the docking protocol.

### 2.2. System Stability

The studied systems, 3CLpro main protease and PLpro papain-like protease in complex with two protein substrates (CS1 and CS2), after 600 ns MD simulations were tested for structural stability using root-mean-square deviation (RMSD) analysis. It is known that the lower the RMSD, the more stable the protein [33]; herein, the 3CLpro and PLpro apo-proteins were considered as control in our MD simulations. The RMSD for the 3CLpro apo-protein was found to increase initially and reached an average of 2.67 Å for the 600 ns simulation; the observed motion between conformations of the 3CLpro main protease suggested the opening and closing of the protease. Similarly, for the PLpro apo-protein, stability was observed during the 600 ns simulation with an average RMSD value of 2.50 Å.

**Figure 3:**
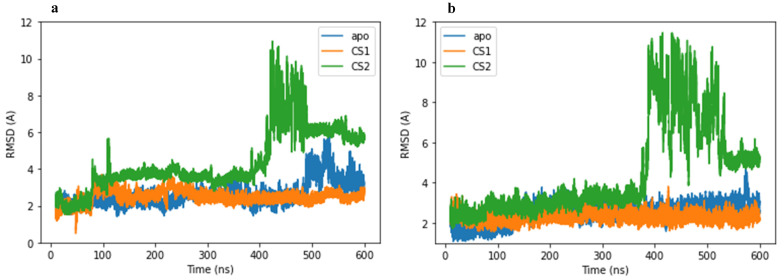
Root-mean-square deviation plot for (**a**) apo-3CLpro protease (blue) and complexes of 3CLpro with CS1 (orange) and CS2 (green) during the 600 ns MD simulation time. (**b**) apo-PLpro protease (blue) and complexes of PLpro with CS1 (orange) and CS2 (green) during the 600 ns MD simulation time.

The RMSD values from MD simulation of the protein–ligand complexes studied were based on the alpha carbon backbone for the protein and the ligand together throughout the trajectory. Hence, the 3CLpro–CS1 complex shows stability throughout the simulation at an average RMSD of 2.50 Å; it is therefore possible that the presence of CS1 induces stability in the 3CLpro protease. It is notable to mention that during the 600 ns simulation, the protein substrate (CS1) maintained a reasonable distance to the active site residues (Cys145/His41), implying that the main protease could play a potential role in the cleavage of the CS1 substrate (Figure 3a and Figure S1). The 3CLpro–CS2 complex reached equilibrium around 100 ns into the simulation, and stability of the α-C backbone was observed for the next 300 ns of simulation with an average RMSD value of 4.35 Å. However, a significant deviation was observed after 400 ns, reaching a maximum value of 10.0 Å, indicating that destabilization occurs at this point in the simulation (Figure 3a). This major deviation is a result of the presence of CS2 protein substrate as evidenced by the lack of this destabilization in the apo-3CLpro case. It is also worth mentioning that the CS2 substrate moves from the active site, and this suggests the unlikelihood of cleavage of the CS2 substrate by 3CLpro in accordance with the literature [5,8] (Figure S2).

In the case of the PLpro–CS1 complex, stability was observed throughout the 600 ns of simulation with no considerable deviation and with an average RMSD value of 2.30 Å. The behavior was similar to the 3CLpro–CS1 complex, where the presence of CS1 again further increased the stability of the PLpro protease. Likewise, during the 600 ns simulation, the protein substrate (CS1) retained proximity to the active site (Cys 113/His 274/Asp 288); this observation supports that cleavage of the substrate CS1 can occur with this protease (Figure 3b and Figure S4). Finally, for the PLpro–CS2 complex, an initial stability evidenced by a constant initial RMSD value of approximately 4.42 Å is apparent; however, a significant deviation occurred around 400 ns with an increased RMSD value of 10.75 Å (Figure 3b). This destabilization was associated with the loss of the CS2 substrate from the active site (Figure S5). Given that there is literature evidence for cleavage of CS2 by PLpro, it may be that the rate of proteolysis by the papain-like protease is different for the cleavage of both CS1 and CS2 since we observe exchange during dynamics of the CS2 substrate. Overall, the protein substrates (CS1 and CS2) behave similarly in both the 3CLpro and PLpro proteases.

Comparison of all the complexes shows that the RMSD of the systems is stable in the region from 100 ns to 400 ns during MD simulations. Therefore, the binding affinity was considered in this region of dynamics for all the complexes.

### 2.3. Structural Flexibility

**Figure 4:**
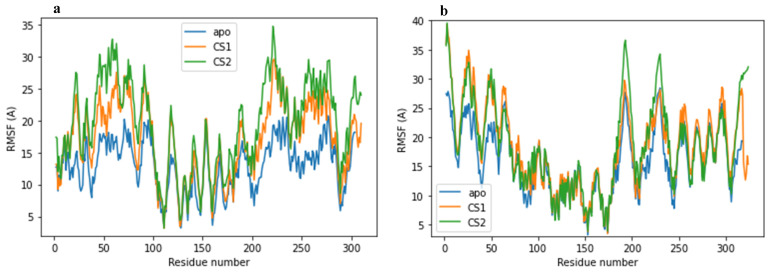
Root-mean-square fluctuation plot for (**a**) apo-3CLpro protease (blue) and complexes of 3CLpro with CS1 (orange) and CS2 (green) during the 600 ns MD simulation time. (**b**) apo-PLpro protease (blue) and complexes of PLpro with CS1 (orange) and CS2 (green) during the 600 ns MD simulation time.

The structural flexibility of the complexes was measured using the root-mean-square fluctuation (RMSF) parameter, defined as the residual fluctuation of every single atom about its average position [34]. All residual fluctuations were examined, with average RMSF values of 12.95, 17.00 and 19.38 Å for apo-enzyme, CS1 and CS2 in complex with the 3CLpro main protease; an average RMSF value of 15.96, 18.44 and 18.78 Å was observed for apo-enzyme, CS1 and CS2 in complex with the PLpro papain-like protease, respectively. Distinct residue fluctuations were observed for the loops and domains of 3CLpro [34] in the presence of the non-structural protein substrates (CS1 and CS2). While the RMSF suggests no significant protein functional difference for the apo-PLpro, CS1 and CS2 complexes. It can be observed from both RMSF graphs that the 3CLpro/PLpro–CS2 exhibits a higher level of fluctuations for both systems. Comparing all four complexes, the predominant motion by which 3CLpro/PLpro proteases could “pull” CS1 and CS2 substrates closer is more pronounced for CS2, indicative of poor/less interaction with their active sites, respectively, and possibly lower binding free energy against CS2 for the PLpro protease. This characteristic was monitored over the simulation time as shown in Figure 4a,b.

### 2.4. Dynamic Cross-Correlation Matrix (DCCM)

**Figure 5:**
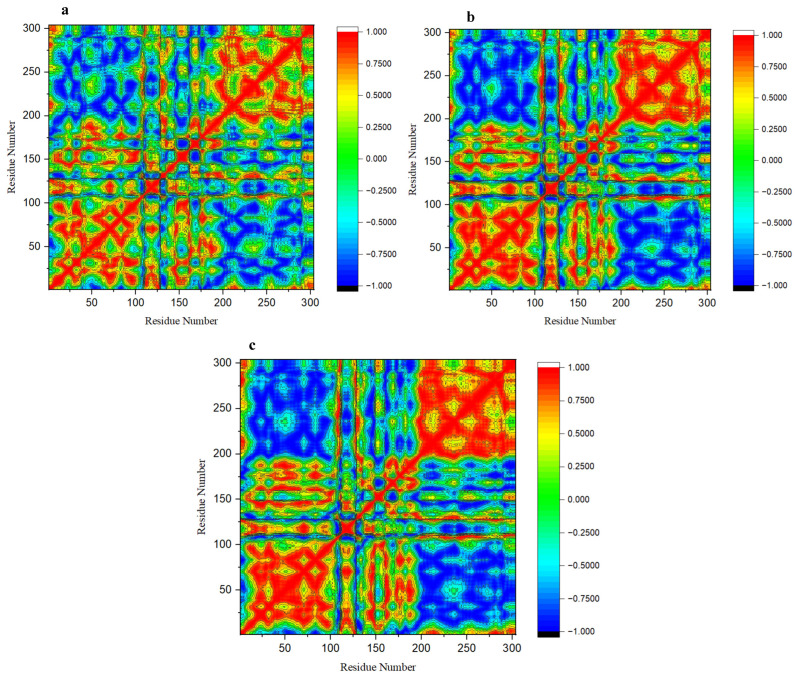
DCCM plot of the alpha carbon fluctuations for the 600 ns simulation period: (**a**) 3CLpro apo-protein; (**b**) 3CLpro–CS1 complex; (**c**) 3CLpro–CS2 complex.

Further analysis of the conformational changes of the 3CLpro/PLpro proteases upon CS1 and CS2 binding was performed by computing the DCCM on the α-carbon atom positions, and this analysis allows the examination of the protein dynamics and available correlated motions. The DCCM of the backbone α-carbon atom variations within the complexes is represented in Figure 5 and Figure S7. The graph shows correlated (highly positive, 0.7500–1.000) and anti-correlated motions (highly negative, −0.7500–−1.000) of specific residues. In the case of 3CLpro protease, it was observed that the presence of both CS1 and CS2 protein substrates affects the DCCM of the 3CLpro apo-protein, where the active site residues are more anti-correlated and other residues of the protease show a more correlated motion. However, for the PLpro protease, the protein substrates (CS1 and 2) have no significant effect on the DCCM of the PLpro apo-protein (Figure S7). This could establish the observations from the literature that the papain-like protease is identified for cleavage at these points [23,26]. However, for the PLpro protease, the substrates (CS1 and 2) have no significant effect on the DCCM of the PLpro apo-protein.

### 2.5. Radius of Gyration

**Figure 6:**
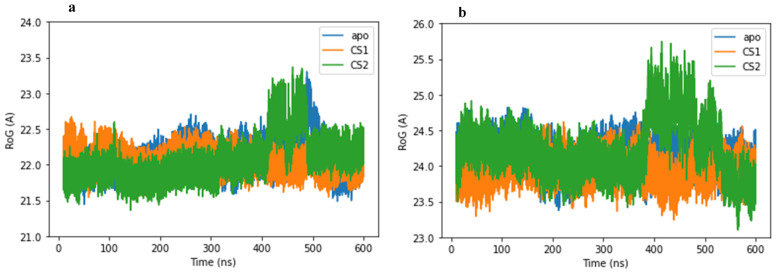
Radius of gyration plot for (**a**) apo-3CLpro protease (blue) and complexes of 3CLpro with CS1 (orange) and CS2 (green) during the 600 ns MD simulation time. (**b**) apo-PLpro protease (blue) and complexes of PLpro with CS1 (orange) and CS2 (green) during the 600 ns MD simulation time.

The radius of gyration (RoG) is a parameter used to determine the structural compactness of the studied systems. RoG values for all complexes (apo-3CLpro/PLpro, 3CLpro/PLpro–CS1 and 3CLpro/PLpro–CS2) are shown in Figure 6a,b. A more compacted and stable structure was observed for the CS1 complex with both 3CLpro and PLpro proteases. RoG values were mostly stable for the first 400 ns MD simulation, which was followed by a slight fluctuation in the presence of CS2 for both 3CLpro and PLpro proteases and then became stable indicating MD simulation reached equilibrium and compact structures. The average values for apo-3CLpro main protease, 3CLpro–CS1 and 3CLpro–CS2 complexes are 22.09, 22.06 and 22.07 Å, respectively, while for the apo-PLpro papain-like protease, PLpro–CS1 and PLpro–CS2 complexes, they were 24.12, 23.95 and 24.21 Å, respectively. The RoG values are approximately similar to each other which clearly indicates that there are only moderate conformational changes during the simulation [35,36]. In addition, the difference in/lower RoG value of 3CLpro/PLpro–CS1 complexes could reveal that the CS1 protein substrate binds better to both proteases than CS2.

### 2.6. Binding Free Energies

The binding affinities of the protein substrates (CS1 and CS2) were estimated toward the 3CLpro main protease and PLpro papain-like protease, in the range of 100 ns to 400 ns (due to reasons mentioned above). The MM/PBSA algorithm evaluates energies through trajectory snapshots extracted from a system MD production run; the estimated energy functions and the binding free energies are shown in Table 3. A highly negative result denotes a favored ligand binding, i.e., the more negative the interacting energy, the better the ligand selectivity and affinity for the protease. Snapshots were taken within the range of 400 ns trajectories to determine the MM/PBSA energies, and it was observed that both CS1 and CS2 have relatively similar binding affinities toward the 3CLpro protease at −10.10/−10.23 kcal mol^−1^. Interestingly, the docking score, RMSD and RMSF analysis indicates a remarkable binding for 3CLpro–CS1. Based on the aforementioned observations, it is possible that the CS2 is binding with strength to a different site from the active site of the 3CLpro main protease, which further establishes the non-possibility of the main protease cleaving the CS2 protein substrate [5,8]. While only CS1 shows a more favored binding affinity toward the PLpro protease with a binding energy value of −15.83 kcal mol^−1^, the difference between binding of the PLpro–CS1 and PLpro–CS2 is −14.31 kcal mol^−1^ which is very high, indicative of the loss of the CS2 protein substrate (less interaction) in the active site. The observed lower RoG values for 3CLpro–CS1 and PLpro–CS1 22.06/23.95 Å, respectively, could potentially indicate a lengthier inhibitor residence time in the binding sites. It can also be seen from Table 3 that the electrostatic contribution is crucial in driving the total binding energies of the complexes in comparison to the van der Waals energies. Likewise, the computed ΔG_gas_ values for all the complexes are high but relatively low (−9.31 kcal mol^−1^) in the 3CLpro–CS1 complex, showing a more hydrophilic interaction, and this could contribute to its binding affinity and possible cleavage of the CS1 by the 3CLpro protease.

Although there is no experimental value for these protein substrates from the literature, the binding energy value for N3 in complex with 3CLpro −42 kJ mol^−1^ (−10.0 kcal mol^−1^) has been estimated theoretically [37], which is comparable with the computed binding energies for 3CLpro–CS1/CS2 in this study.

### 2.7. Hydrogen Bonds

**Figure 7:**
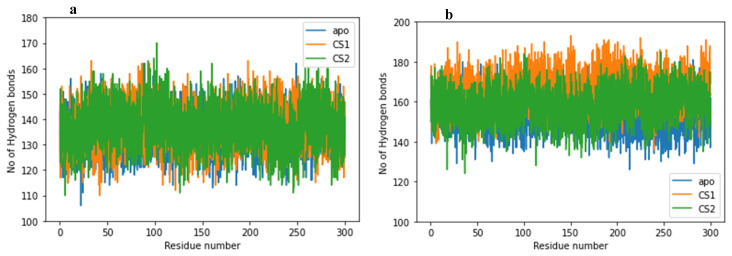
Hydrogen bond formation plot for (**a**) apo-3CLpro protease (blue) and complexes of 3CLpro with CS1 (orange) and CS2 (green) during the 600 ns MD simulation time. (**b**) apo-PLpro protease (blue) and complexes of PLpro with CS1 (orange) and CS2 (green) during the 600 ns MD simulation time.

Hydrogen bonds promote molecular interaction, protein–ligand interaction and binding. The presence of hydrogen bond formation between amino acid residues is an important factor in determining the stability of the amino acids. Therefore, we measured the hydrogen bond formed during the 600 ns MD simulation time and graphically represent it in Figure 7a,b.

The 3CLpro apo-protein, 3CLpro–CS1 and 3CLpro–CS2 complexes exhibit a relative average H-bond formation of 135.66, 136.92 and 137.18, respectively. The higher average H-bond formation observed for the 3CLpro–CS2 is comparable to its binding energy where the CS2 is predicted to bind with strength to a different site of the 3CLpro main protease, whereas the PLpro apo-protein, PLpro–CS1 and PLpro–CS2 complexes show an average H-bond formation of 153.82, 165.79 and 158.23, respectively. The relative decrease in the H-bond formation for the PLpro–CS2 could be a response from the loss of the CS2 from the active site which invariably affects its binding strength. H-bond reduction could result in structural destabilization and conformational changes which affect the binding affinities of drug/protein substrates. To further assess the relative stability of the studied complex systems, the H-bond distances and occupancy were monitored throughout the simulation time, and the results are presented in Table 4.

In the 3CLpro–CS1 system, CYS 144 exhibits H-bond occupancy of 54.42% with an average H-bond distance of 2.87 Å at an angle of 157.75°, while CYS_144 in 3CLpro–CS2 exhibits a higher H-bond occupancy of 56.41% with an average H-bond distance of 2.88 Å at an angle of 158.46°. In contrast, HIE 40 in 3CLpro–CS1 shows 28.89% higher H-bond occupancy, with a relative H-bond distance of 2.91 Å at an angle of 154.64°, and 3CLpro–CS2 shows a 15.23% H-bond occupancy with an H-bond distance of 2.92 Å at an angle of 156.07°. Both HIS 40 and CYS 144 are prime active site residues in 3CLpro, which contributes effectively to the ligand binding having high H-bond occupancy (Table 4).

However, for the active site residues CYS 113, HIS 274 and ASP 288 in the SARS-CoV-2 PLpro for CS1 substrate, we record an H-bond occupancy of 75.14% (2.83 Å—H-bond distance), 28.32% (2.91 Å—H-bond distance) and 42.95% (2.82 Å—H-bond distance), respectively, while for CS2 substrate, CYS 113, HIS 274 and ASP 288 exhibit an H-bond occupancy of 77.47% (2.79 Å—H-bond distance), 35.22% (2.90 Å—H-bond distance) and 44.35% (2.82Å—H-bond distance), respectively (Table 4). The different H-bond occupancy and angle recorded for the different residues of the protease substrates (CS1 and CS2) in the various active site conformational space could be responsible for their binding energies, which eventually determine the mechanism of proteolysis.

## 3. Materials and Methods

### 3.1. System and Ligand Preparations

**Figure 8:**
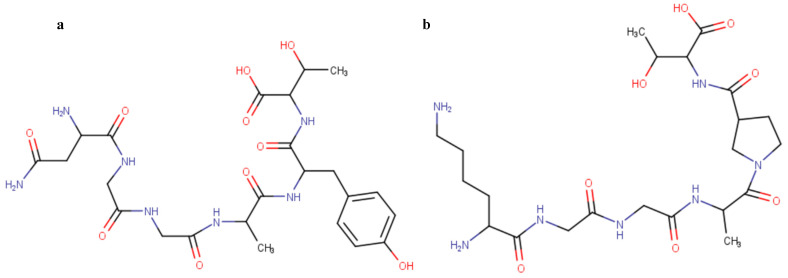
Two-dimensional structures of the non-structural protein cleavage points (**a**) CS1 and (**b**) CS2 used in the study.

The X-ray co-crystal structure of 3CLpro main protease (PDB ID: 6XA4) [18] and PLpro protease (PDB: 6WX4) [19] were obtained from Protein Data Bank (PDB) at 1.41 Å and 1.66 Å resolutions for molecular docking calculations. The proteins were prepared by isolating the co-crystalized ligands and water molecules present using the graphical user interface (GUI) of BIOVIA Discovery Studio [20]; in the case of 3CLpro, chain A was isolated for docking. The enzyme was saved in PDBQT format after the addition of hydrogen atoms. The protein substrate sequences (CS1 and CS2) were built from a Jupyter notebook, and the structures were optimized within the notebook using the Universal forcefield (UFF) [38]; these were also converted to PDBQT format after the addition of hydrogen atoms so as to be ready to be used for molecular docking (Figure 8).

### 3.2. Molecular Docking

The molecular docking procedure for all complexes (3CLpro/PLpro–CS1 and 3CLpro/PLpro–CS2) considered in this study involves the pose assessment and binding energy prediction using Autodock Vina 1.2.0 [32]. The Gasteiger partial charges algorithm [39] was used to assign atomic charges to both the ligand and protein from AutoDock that is provided by MGL tools 1.2.0 while utilizing AutoDock atom types for outlining both the ligand and protein. The grid box was set with centers x = 20.29 Å, y = 73.09 Å and z = 13.05 Å for PLpro protease and x = 11.06 Å, y = −0.36 Å and z = 22.24 Å for 3CLpro protease, while a search space of 40 Å was set for all (x, y, z) dimensions, and the Vina exhaustiveness was set to 4000. The selected dimensions allowed for the peptide structures of CS1 and CS2 to be contained in the protein complex. Several docking runs were performed using the Lamarckian genetic algorithm [39] in AutoDock Vina 1.2.0 to assess reproducibility of the docking procedure. The docked conformations with the stable and highest binding energies (−9.1 kcal mol^−1^ for CS1 and −6.7 kcal mol^−1^ for CS2) were selected for molecular dynamics (MD) calculations involving the 3CLpro main protease, while the highest and stable docked conformation for CS1 −7.1 kcal mol^−1^ and CS2 −6.8 kcal mol^−1^, respectively, were selected for the MD calculations complex with the PLpro protease. The docking process was validated by re-docking the co-crystalized inhibitor from PDB which was then superimposed on the native co-crystal protein complexes from PDB.

### 3.3. Molecular Dynamics Simulations

Before performing MD simulations, the H^++^ server [40] was used in assigning protonation states for the protein. The restrained electrostatic potential (RESP) method was used to fit the charges of the protein substrate (CS1 and CS2) at the HF/6-31G* level of theory/basis set using Gaussian16 package [41]. Afterward, MD simulations were carried out by adding missing atoms to the complex (SARS-CoV-2 3CLpro/PLpro–CS1 and SARS-CoV-2 3CLpro/PLpro–CS2) systems using the Leap module implemented in the AMBER 18 MD package [42]. All systems considered in this study were solvated using an octahedral TIP3P water box [43], extending at 12 Å outside the protein on all sides to maintain a constant and acceptable system solvation throughout the simulation. Counterions were added and positioned around the protein to ensure the system was neutralized, and both the protein and natural substrate ligands were described using the AMBER force field 16SB [44,45].

The complexes were first energy-minimized at 5000 minimization steps of each steepest descent and conjugate gradient methods, while a restraint of 20 kcal mol^−1^.Å^−2^ was applied to the backbone atoms of both protein and natural substrate peptide residues. Gradual heating of the system was performed from 0 to 300 K using the Langevin thermostat [46] at a constant volume over 1000 ps, with 10 kcal mol^−1^.Å^−2^ restraints on the backbone atoms. Subsequently, equilibration of the systems was performed at a constant pressure for 1000 ps at 300 K, with harmonic restraint of 5 kcal mol^−1^.Å^−2^, which was later relaxed using weak restraints of 2 kcal mol^−1^.Å^−2^ on only the backbone atoms. All the simulation steps which involve partial and full minimizations, heating and equilibration utilized a non-bonded cutoff of 8 Å. Particle mesh Ewald (PME) was used in calculating the long-range Coulomb forces, and the time step was set to 2 fs, while the SHAKE algorithm [47] was used to constrain the bond length between the heavy atoms and hydrogen atoms to its equilibrium value during the MD simulations. Post dynamics analysis, which includes RMSD, RMSF, RoG, hydrogen bonding and DCCM, was performed after the MD simulations using CPPTRAJ modules in AMBER 18. The trajectories were visualized using Chimera 1.16 software [48], and plots were generated using Matplotlib graphics [49].

### 3.4. Post-Simulation Analysis and Thermodynamics Calculation

#### 3.4.1. System Stability

The system stability is assessed by evaluating the conformational backbone changed via the root-mean-square deviation (RMSD) calculation. The RMSD trajectory of the protein backbone alpha carbon (Cα) was generated with the CPPTRAJ module according to Equation (1) (Equation (1)).

(1) $$RMSD\left(v,w\right)=\sqrt{\frac{1}{n}\overset{n}{\underset{i=1}{\sum }}||v_{i}-w_{i}}|{|}^{2}\;$$

In Equation (1), the standard deviation between two amino acids *v* and *w* is calculated according to the interatomic distance where *v*_i_ and *w*_i_ are the coordinates of Cα atom in *v* and *w* at the time *i*, respectively, over *n* frames. To measure the molecular stability of biological systems, the radius of gyration was employed, which is the moment of Cα atom inertia from its center of mass. The RoG is the square root of the inertia moment (*I*) divided by mass (m) Equation (2). The parameter was generated using the CPPTRAJ [50] module implemented in the AMBER 18 suite.

(2) $$I=mR^{2}\;$$

#### 3.4.2. Dynamics Conformation

The root-mean-square fluctuation (RMSF) and dynamic cross-correlation matrix (DCCM) were estimated for the 3CLpro/PLpro–CS1 and 3CLpro/PLpro–CS2 complexes. This analysis facilitates the study of the conformational changes induced by the protein substrate (CS1 and CS2) binding. The RMSF prediction approach followed by the CPPTRAJ module is represented in Equation (3), where *x_i_*_(*j*)_ denotes the *i*th Cα atom position in the *j*th model structure, and (*x_i_*) represents the averaged location of the *i*th Cα backbone atom in all models.

(3) $$RMSF=\sqrt{\frac{1}{n}\overset{n}{\underset{j}{\sum }}|x_{i\left(j\right)}-(x_{i}}){|}^{2}\;$$

Cross-correlation of protein residues is predicted through 3D contour graphics that show time-correlated data across the residues [51]. To examine the dynamics induced by CS1 and CS2, the DCCM was estimated. This analysis helps in predicting the cross-correlated shifts that occur in the protein Cα backbone (Equation (4)), and outputs were generated using the CPPTRAJ module available in the AMBER 18 program.

(4) $$C_{ij}=\langle \Delta r_{i}\;*\;\Delta r_{j}\rangle /{\left(\langle {\Delta r}_{i}^{2}\rangle \langle {\Delta r}_{j}^{2}\rangle \right)}^{1/2}\;$$

Note that ∆*r_i_* and ∆*r_j_* symbolize the shift of *i*th and *j*th atoms from the average, respectively, while *i* and *j* denote the *i*th and *j*th Cα residues. The *C_ij_* parameter ranges from −1 to +1, and the higher and lower bounds indicate the best correlated (+) and anti-correlated (−) motions for the simulation.

#### 3.4.3. Binding Free Energy Calculations

To provide detailed information on the interaction between the enzyme and protein substrate, the binding energies were calculated, and this was achieved by utilizing the molecular mechanism integrated with the Poisson–Boltzmann (MM-PBSA) method [52]. From the MM-PBSA approach, the binding free energies were obtained for both 3CLpro/PLpro–CS1 and 3CLpro/PLpro–CS2 complexes. This analysis provides a better understanding of various energy contributions such as entropy and enthalpy of the molecular recognition [52,53]. For 600 ns trajectories, we considered 1000 snapshots during the binding free energy calculation. Equations (5)–(8) below are used to describe the enzyme–substrate binding free energy (∆G_bind_) and its components for each molecular species (complex, protein and ligand).

(5) $$\Delta G_{\mathrm{bind}}=G_{\mathrm{complex}}-G_{\mathrm{receptor}}-G_{\mathrm{ligand}}$$

(6) $$\Delta G_{\mathrm{bind}}=E_{\mathrm{gas}}+G_{\mathrm{sol}}-\mathrm{TS}\;$$

(7) $$E_{\mathrm{gas}}=E_{\mathrm{int}}+E_{\mathrm{vdW}}+E_{\mathrm{ele}}$$

(8) $$G_{\mathrm{sol}}=G_{\mathrm{GB}}+G_{\mathrm{SA}}$$

In Equations (5)–(8), the parameters E_gas_, E_int_, E_ele_ and E_vdw_ denote the gas-phase energy, internal energy, Coulombs’ energy and van der Waals energy, respectively. E_gas_ is directly parametrized from the FF14SB forcefield terms. The solvation free energy (G_sol_), which is evaluated from the polar states (G_GB_) and non-polar states energy (G_SA_), provides a quantitative analysis of the explicit water that is contributing to the binding process, while parameters T and ∆S are the temperature and the total solute entropy, respectively. Predicting the active residue contribution was also made possible by the binding free energy method of MM/PBSA.

## 4. Conclusions

In this study, we used a combination of molecular dockings and MD simulation to provide a broad insight into the dynamics of non-structural protein (CS1 and CS2) against SARS-CoV-2 3CLpro and PLpro proteases. This protocol allows a comparative study of the recognition of the protein substrate (CS1 and CS2). Post molecular dynamics analyses, such as thermodynamics calculations, DCCM analysis and hydrogen bond occupancy, were performed which provided a range of information on the binding impact of both CS1 and CS2 substrates on 3CLpro and PLpro proteases, respectively.

The molecular docking analysis showed that CS1 has the highest binding affinity of −9.1 kcal mol^−1^ and −7.1 kcal mol^−1^ for 3CLpro and PLpro protease, respectively. The MD simulations enabled us to determine the binding conformation of the investigated protein substrate, and it was observed that there exists a possibility of the 3CLpro protease cleaving the CS1 substrate (due to the strength of interaction) and not the CS2 protein substrate (this second observation supports reports in the literature). For the former observation, exploration of the proteolysis mechanism of CS1 by 3CLpro using QM/MM techniques will be necessary to explain why it has not been observed experimentally.

On the other hand, the cleavage of CS2 by PLpro is observed experimentally, yet exchange is observed during dynamics. It could be that the rate of proteolysis of CS2 by PLpro protease is very fast, followed by a rapid loss of the CS2 substrate, which is supported by the computed free binding energies. The binding energy and hydrogen bond formation for the 3CLpro–CS2 system is higher compared to the 3CLpro–CS1 complex, and this may be due to the difference in binding for the CS2 protein substrate, supported by the difference in distance between the substrate and protease during both dynamics simulations (see Supplementary Material). Based on the parameters studied herein, CS1 substrate exhibits favorable binding to 3CLpro/PLpro protease from the docking score and high stability through the RMSD, RMSF and RoG plots.

In summary, the findings provide an important insight that could help in the development of novel SARS-CoV-2 3CLpro inhibitors by mimicking the CS1 protein substrate; such an inhibitor could act on both 3CLpro and PLpro. Further QM/MM investigations of the proteolysis mechanism will provide insight as to why 3CLpro does not cleave CS1 experimentally.

## Figures and Tables

**Table 1:** List of non-structural proteins cleaved by PLpro and 3CLpro, respectively, with * marking the cleavage point.

Proteolytic Cleaved Sites by PLpro	Non-Structural Protein ID
Asn-Gly-Gly*Ala-Tyr-Thr	Nsp1-Nsp2 (CS1)
Lys-Gly-Gly*Ala-Pro-Thr	Nsp2-Nsp3 (CS2)
Lys-Gly-Gly*Lys-Iso-Val	Nsp3-Nsp4
Proteolytic cleaved sites by 3CLpro	
Val-Leu-Gln*Ser-Gly-Phe	Nsp4-Nsp5
Thr-Phe-Gln*Ser-Ala-Val	Nsp5-Nsp6
Thr-Val-Gln*Ser-Lys-Met	Nsp6-Nsp7

**Table 2:** Root-mean-square values (in Å), or overlay similarity of the superimposed amino peptide residues of re-docked complexes with native and docked complexes considered in this study.

No	Complexes	RMSD Value (Å)
	3CLpro	
1	6XA4 PDB vs. re-docked 6XA4	0.86 (RMSD)
2	6XA4 PDB vs. 3CLpro–CS1	0.77 (overlay similarity)
3	6XA4 PDB vs. 3CLpro–CS2	0.78 (overlay similarity)
	PLpro	
6	6WX4 PDB vs. re-docked 6WX4	0.83 (RMSD)
7	6WX4 PDB vs. PLpro–CS1	0.74 (overlay similarity)
8	6WX4 PDB vs. PLpro–CS2	0.73 (overlay similarity)

**Table 3:** Calculated binding free energies and their corresponding components (in kcal mol^−1^) for the complexes of 3CLpro/PLpro with CS1 and CS2 using the AMBER18 package.

System	ΔE_vdw_	ΔE_ele_	ΔG_gas_	ΔG_polar_	ΔG_nonpolar_	ΔG_sol_	ΔG_bind_
3CLpro							
CS1	−9.97	−75.60	−9.31	1.21	−2.00	−0.79	−10.10
CS2	−8.09	−133.21	−68.13	60.05	−2.16	57.90	−10.23
PLpro							
CS1	−15.96	−154.29	−95.66	82.71	−2.87	79.83	−15.83
CS2	−4.24	−148.23	−80.12	79.69	−1.09	78.60	−1.52

Note: ΔE_vdw_ = van der Waals energy, ΔE_ele_ = electrostatic energy, ΔG_bind_ = total binding energy, ΔG_solv_ = solvation free energy.

**Table 4:** Hydrogen bonds between the complexes of 3CLpro/PLpro with CS1 and CS2 over the simulation time.

System 3CLpro	Acceptor	Donor	Occupancy (%)	Distance (Å)	Angle (°)
CS1	CYS_144@O	ASN_27@H-N	54.42	2.8722	157.7537
	GLY_307@O	GLU_165@H-N	33.42	2.8901	162.1835
	HIE_40@O	CYS_43@H-N	28.89	2.9051	154.6399
	THR_310@O	THR_189@H-N	26.29	2.8622	158.9503
	CYS_144@O	ASN_27@HD22-ND2	25.39	2.8489	158.2798
	THR_310@O	ALA_190@H-N	18.73	2.8603	159.0912
	TYR_309@O	GLN_188@HE22-N322	15.13	2.8431	159.6982
	THR_310@OXT	ALA_190@H-N	12.56	2.8654	157.2788
	THR_310@OXT	THR_189@H-N	9.63	2.8664	153.5904
	GLY_307@O	TRY_309@H-N	7.66	2.9030	146.1381
	TYR_309@O	THR_310@HG1-OG1	6.96	2.8039	161.6148
	ASN_305@O	ALA_308@H-N	4.47	2.9094	152.8739
	ASN_305@OD1	GLY_306@H-N	3.70	2.8340	144.2798
	ASN_305@O	SER_45@HG-OG	3.40	2.7780	159.0601
	ALA_308@O	ASN_305@H1-N	3.00	2.8331	153.1770
CS2	CYS_144@O	ASN_27@H-N	56.41	2.8798	158.4557
	GLY_306@O	SER_138@HG-OG	43.59	2.7128	161.2717
	THR_310@O	ARG_3@HH21-NH2	28.06	2.8006	158.7969
	THR_310@OXT	ARG_3@HH21-NH2	18.33	2.8087	156.1145
	HIE_40@O	CYS_43@H-N	15.23	2.9166	156.0746
	HIE_40@ND1	HIE_40@H-N	12.30	2.9060	141.5077
	PRO_309@O	THR_310@HG1-OG1	2.97	2.8355	159.3578
	ALA_308@O	LEU_140@H-N	1.93	2.8732	154.1803
PLpro					
CS1	CYS_113@O	THR_117@HG1-OG1	75.14	2.8348	158.5441
	THR_324@O	GLY_273@H-N	71.53	2.8464	161.7714
	ASN_319@OD1	TYR_323@H-N	54.02	2.8828	162.5650
	ASP_288@OD2	HIE_274@HE2-NE2	42.95	2.8187	157.9014
	CYS_113@O	THR_117@H-N	39.45	2.9013	159.4459
	ASN_319@OD1	ALA_322@H-N	38.32	2.8724	153.1878
	ASP_288@O	LEU_291@H-N	32.42	2.9040	159.4916
	THR_324@OXT	ASN_111@HD22-ND2	31.66	2.8407	149.4410
	HIE_274@O	THR_267@H-N	28.32	2.9130	162.4926
	TRY_323@O	ASN_319@H1-N	13.06	2.7518	145.6913
	GLY_20@O	TYR_270@HH-OH	4.47	2.7677	161.4565
	ASN_319@OD1	GLY_320@H-N	4.23	2.8071	144.6121
CS2	CYS_113@O	THR_117@HG1-OG1	77.47	2.7872	162.2125
	ASP_288@OD2	HIE_274@HE2-NE2	44.35	2.8228	160.2619
	CYS_113@O	THR_117@H-N	38.59	2.9075	159.0521
	ASP_288@OD1	HIE_274@HE2-NE2	37.65	2.8263	159.7712
	HIE_274@O	THR_267@H-N	35.22	2.9013	161.0575
	ASP_288@O	LEU_291@H-N	30.36	2.9058	158.9475
	PRO_323@O	ARG_168@HH12-NH1	6.83	2.8173	156.2786
	PRO_323@O	THR_324@HG1-OG1	6.70	2.8011	162.3707
	THR_324@O	THR_324@HG1-OG1	4.63	2.7763	146.7643
	THR_324@OG1	ARG_168@HH22-NH2	2.83	2.8560	155.1610
	GLY_320@O	ARG_168@HH22-NH2	2.30	2.8517	150.3676
	PRO_318@OXT	LYS_219@HZ1-NZ	2.17	2.7873	159.4122
	LYS_319@O	GLN_176@HE21-NE2	2.10	2.8605	158.4366

## Supplementary Materials

The following supporting information can be downloaded at: https://www.mdpi.com/article/10.3390/molecules27238251/s1. The distance plots and 3D structures generated from chimera for the SARS-CoV-2 3CLpro/PLpro complexed with the protein substrates (CS1 and 2) are provided in the supplementary information.
