# Supplementary material for: Insights into the Dynamics and Binding of Two Polyprotein Substrate Cleavage Points in the Context of the SARS-CoV-2 Main and Papain-like Proteases
Source: Molecules. 2022 Nov 26;27(23):8251. doi: 10.3390/molecules27238251 (PMC9740519; doi:10.3390/molecules27238251)
Supplement: Supplementary file 1 [file molecules-27-08251-s001.zip › molecules-1968827-supplementary.pdf]

Supplementary Information

Insights into the Dynamics and Binding of two Polyprotein Substrate Cleavage Points in the context of the SARS-CoV-2 Main and Papain-like Proteases

Zainab Kemi Sanusi [1] and Kevin Alan Lobb [1,2,*]

[1] Department of Chemistry, Rhodes University, Makhanda 6140, South Africa; sanusizainab10@gmail.com
[2] Research Unit in Bioinformatics (RUBi), Rhodes University, Makhanda 6140, South Africa
* Correspondence: k.lobb@ru.ac.za

Table of Contents

Figure S1(a). Distance plot of the CS1 protein substrate complex with 3CLpro main protease. 2

Figure S1(b). 3D picture from chimera showing the distance of the CS1 substrate from the active site of the 3CLpro main protease. **Error! Bookmark not defined.**

Figure S2(a). Distance plot of the CS2 protein substrate complex with 3CLpro main protease. 3

Figure S2(b). 3D picture from chimera showing the distance of the CS2 from the active site of the 3CLpro main protease. 3

Figure S3. RMSD plot of the apo 3CLpro main protease plot from Chimera. 4

Figure S4(a). Distance plot of the CS1 protein substrate complex with PLpro papain-like protease. 4

Figure S4(b). 3D picture from chimera showing the distance of the CS1 substrate from the active site of the PLpro protease. 5

Figure S5(a). Distance plot of the CS2 protein substrate complex with PLpro papain-like protease. 5

Figure S5(b). 3D picture from chimera showing the distance of the CS2 from the active site of the PLpro protease. 6

Figure S6. RMSD plot of the apo PLpro papin-like protease plot from Chimera. 6

Figure S7. DCCM plot of the alpha-Carbon fluctuations for the 600 ns simulation period (a) PLpro apo-protein (b) PLpro—CS1 complex (c) PLpro—CS2 complex. 7

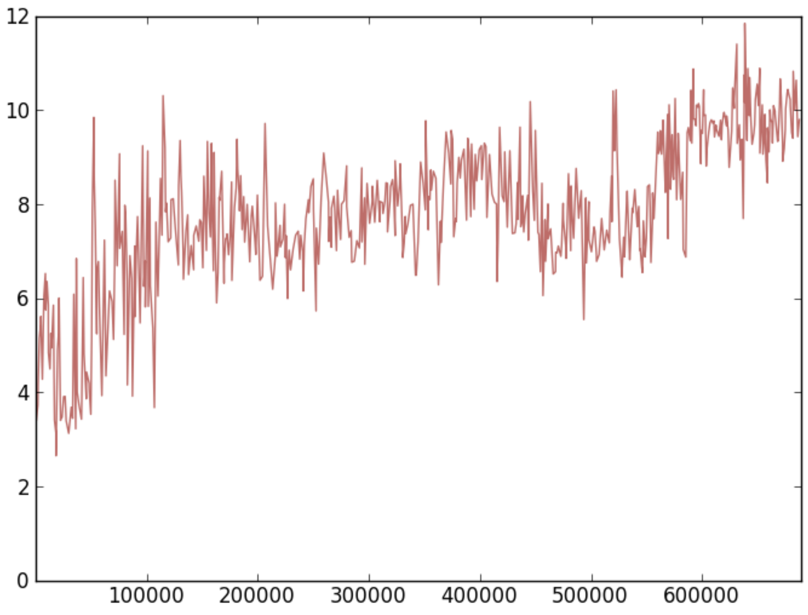

Figure S1(a). Distance plot of the CS1 protein substrate complex with 3CLpro main protease.

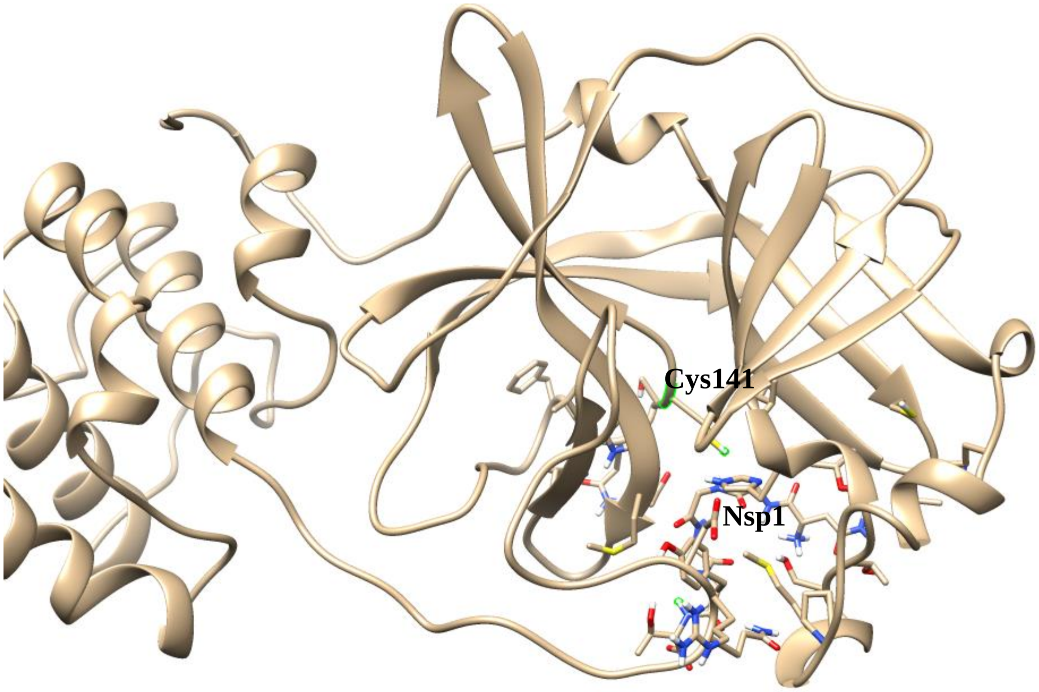

Figure S1(b). 3D picture from chimera showing the distance of the CS1 substrate from the active site of the 3CLpro main protease.

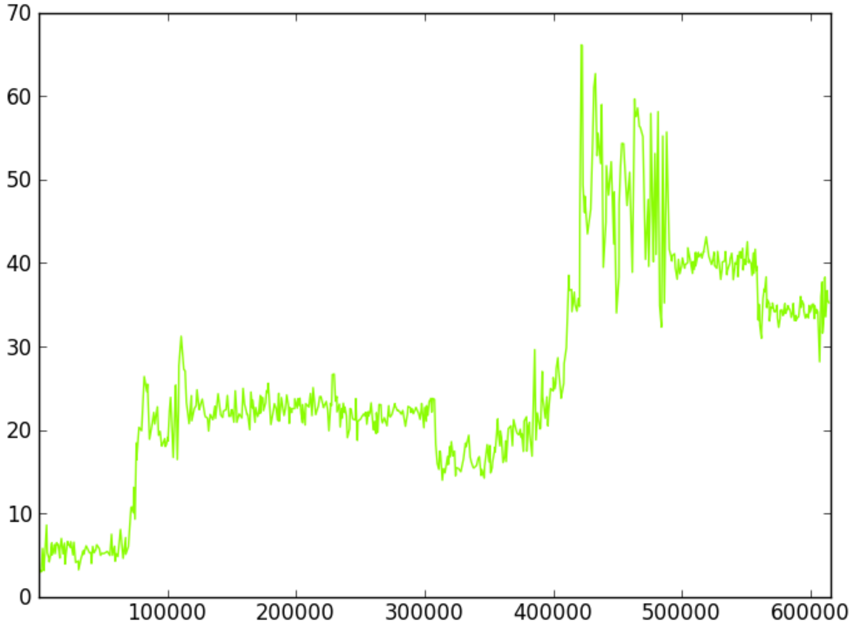

Figure S2(a). Distance plot of the CS2 protein substrate complex with 3CLpro main protease.

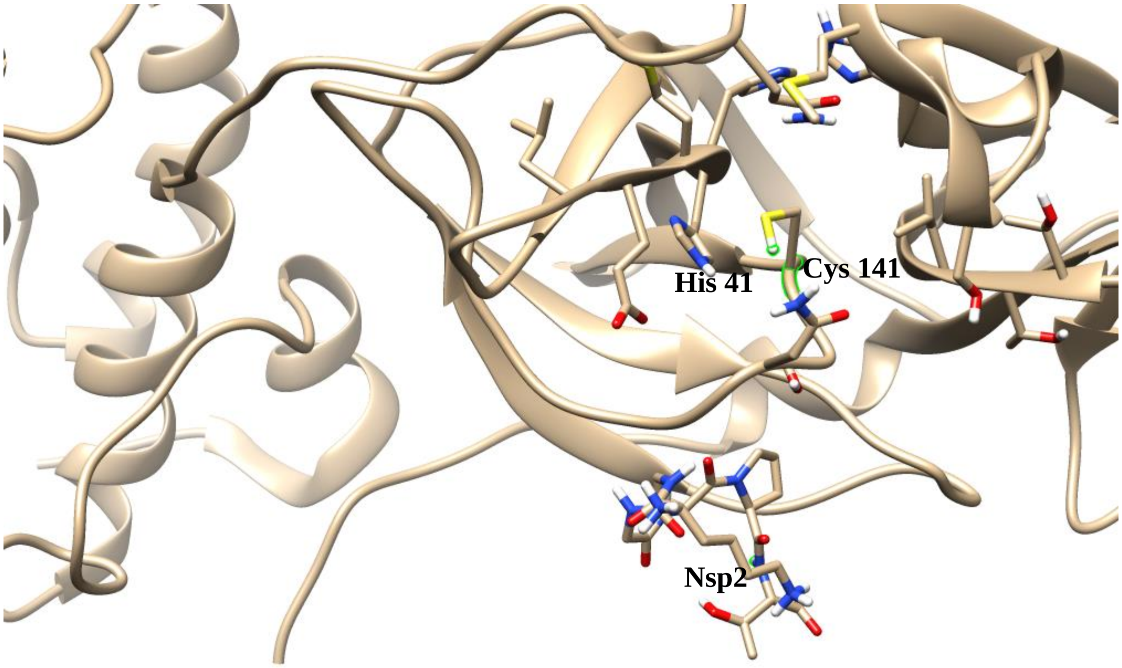

Figure S2(b). 3D picture from chimera showing the distance of the CS2 from the active site of the 3CLpro main protease.

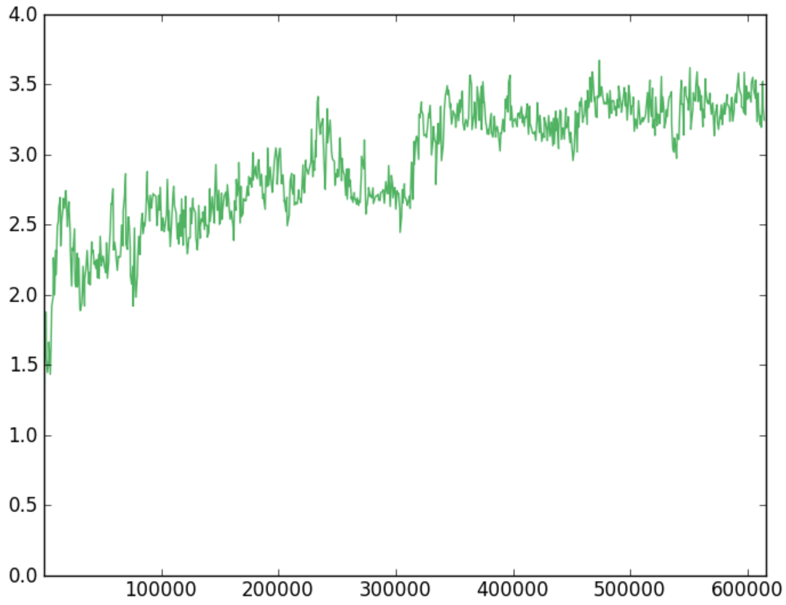

Figure S3. RMSD of the apo 3CLpro main protease plot from Chimera.

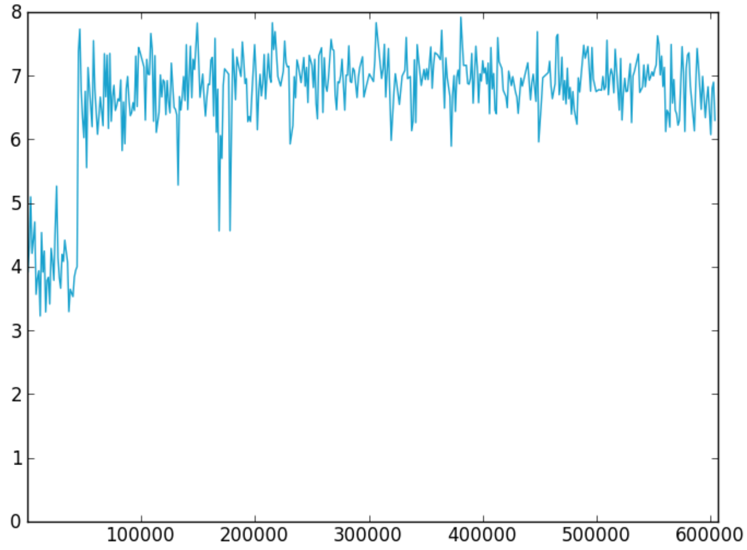

Figure S4(a). Distance plot of the CS1 protein substrate complex with PLpro papain-like protease.

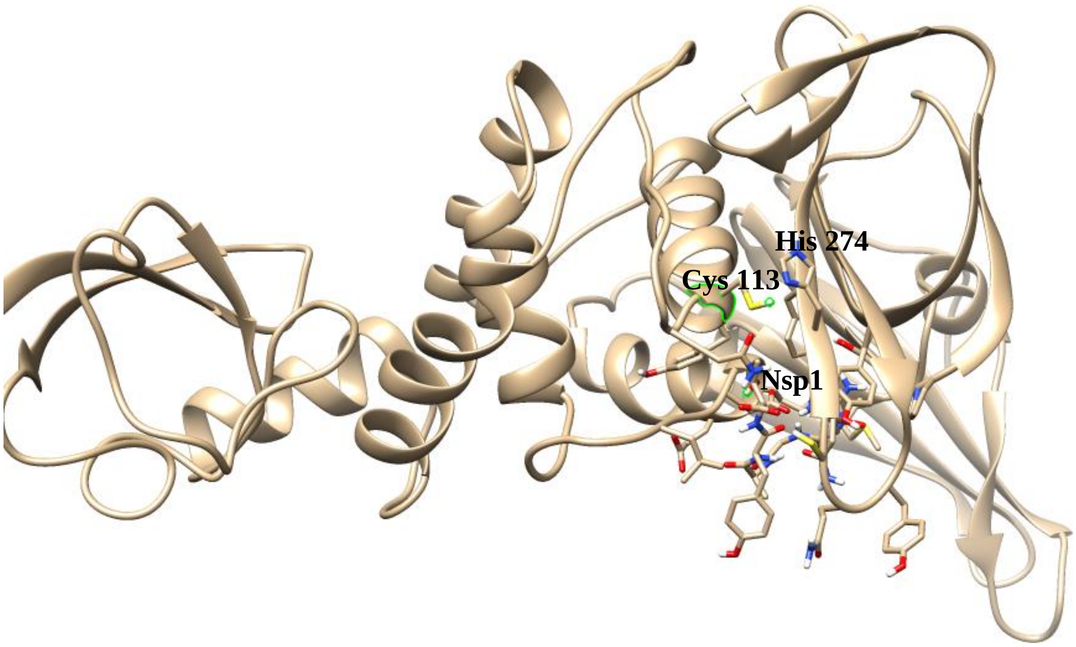

Figure S4(b). 3D picture from chimera showing the distance of the CS1 substrate from the active site of the PLpro protease.

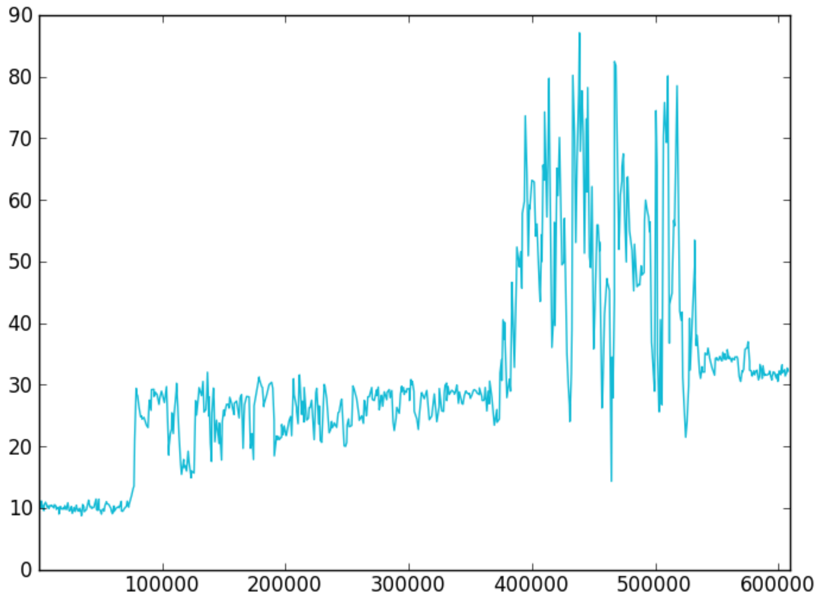

Figure S5(a). Distance plot of the CS2 protein substrate complex with PLpro papain-like protease.

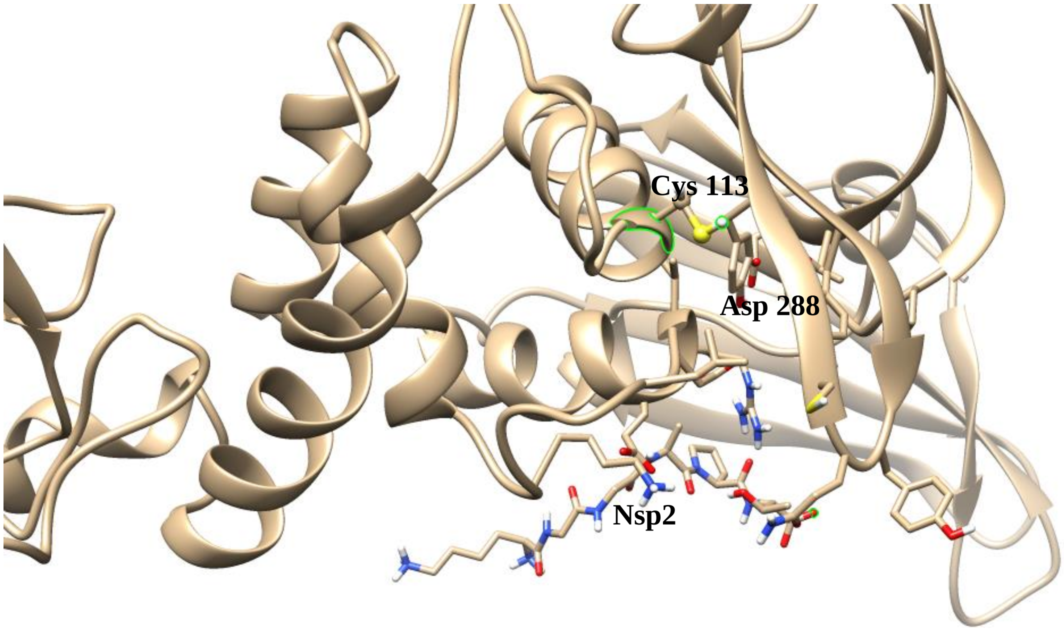

Figure S5(b). 3D picture from chimera showing the distance of the CS2 from the active site of the PLpro protease.

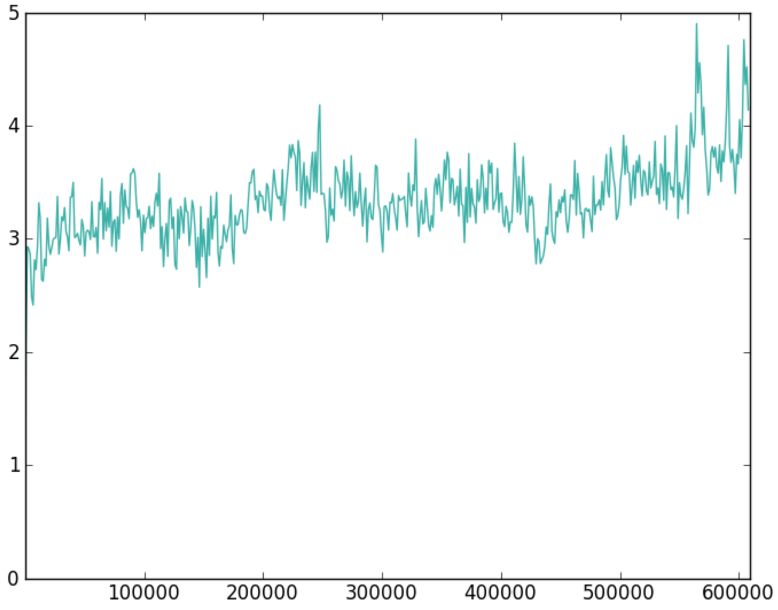

Figure S6. RMSD of the apo PLpro papain-like protease plot from Chimera.

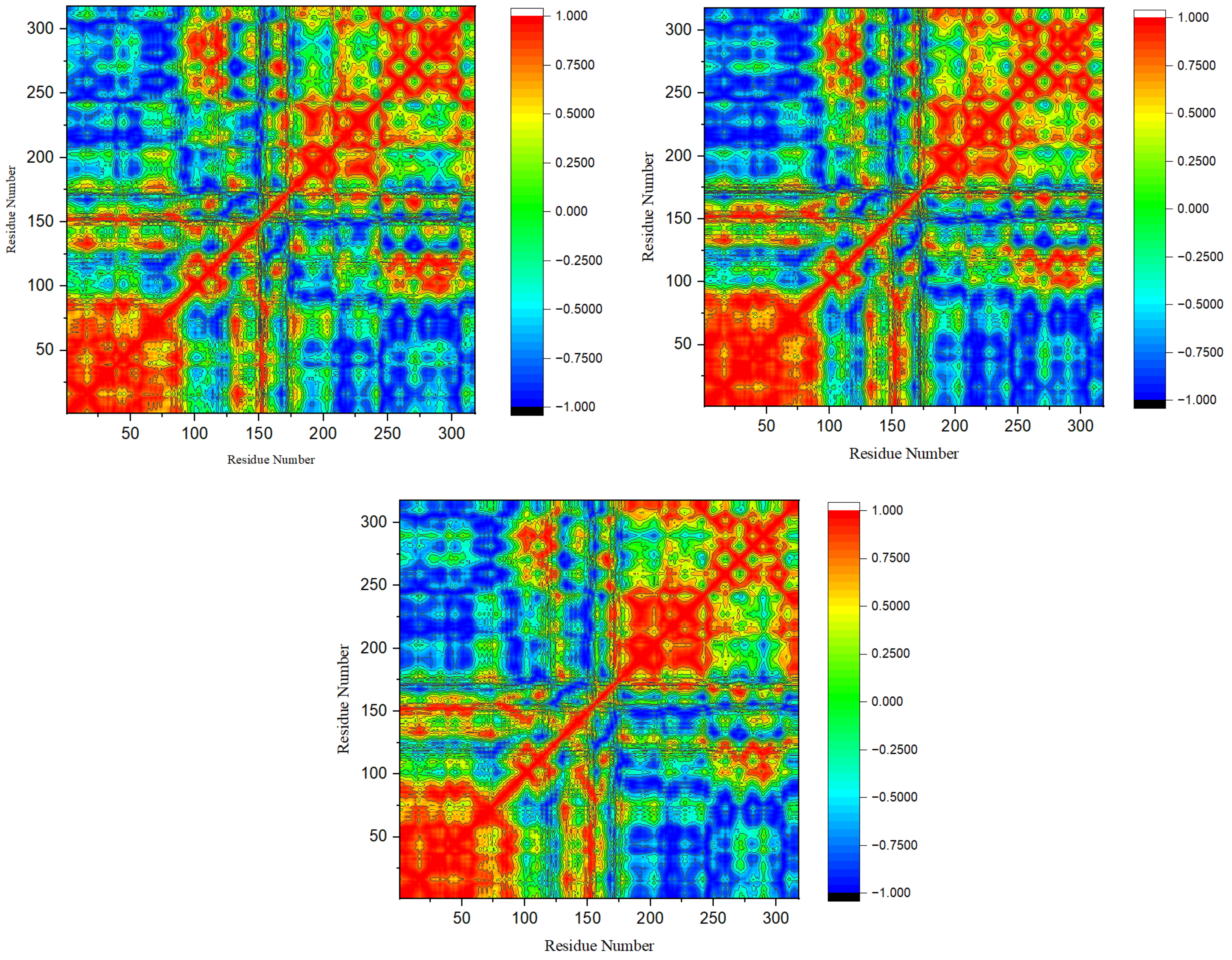

Figure S7. DCCM plot of the alpha-Carbon fluctuations for the 600 ns simulation period (a) PLpro apo-protein (b) PLpro—CS1 complex (c) PLpro—CS2 complex.
